# Health characteristics and consultation patterns of people with intellectual disability: a cross-sectional database study in English general practice

**DOI:** 10.3399/bjgp16X684301

**Published:** 2016-02-24

**Authors:** Iain M Carey, Sunil M Shah, Fay J Hosking, Stephen DeWilde, Tess Harris, Carole Beighton, Derek G Cook

**Affiliations:** Population Health Research Institute, St George’s, University of London, London.; Population Health Research Institute, St George’s, University of London, London.; Population Health Research Institute, St George’s, University of London, London.; Population Health Research Institute, St George’s, University of London, London.; Population Health Research Institute, St George’s, University of London, London.; Faculty of Heath Social Care and Education, Kingston and St George’s, University of London, London.; Population Health Research Institute, St George’s, University of London, London.

**Keywords:** chronic disease, continuity of care, intellectual disability, learning disabilities, primary care

## Abstract

**Background:**

People with intellectual disability (ID) are a group with high levels of healthcare needs; however, comprehensive information on these needs and service use is very limited.

**Aim:**

To describe chronic disease, comorbidity, disability, and general practice use among people with ID compared with the general population.

**Design and setting:**

This study is a cross-sectional analysis of a primary care database including 408 English general practices in 2012.

**Method:**

A total of 14 751 adults with ID, aged 18–84 years, were compared with 86 221 age-, sex- and practice-matched controls. Depending on the outcome, prevalence (PR), risk (RR), or odds (OR) ratios comparing patients with ID with matched controls are shown.

**Results:**

Patients with ID had a markedly higher prevalence of recorded epilepsy (18.5%, PR 25.33, 95% confidence interval [CI] = 23.29 to 27.57), severe mental illness (8.6%, PR 9.10, 95% CI = 8.34 to 9.92), and dementia (1.1%, PR 7.52, 95% CI = 5.95 to 9.49), as well as moderately increased rates of hypothyroidism and heart failure (PR>2.0). However, recorded prevalence of ischaemic heart disease and cancer was approximately 30% lower than the general population. The average annual number of primary care consultations was 6.29 for patients with ID, compared with 3.89 for matched controls. Patients with ID were less likely to have longer doctor consultations (OR 0.73, 95% CI = 0.69 to 0.77), and had lower continuity of care with the same doctor (OR 0.77, 95% CI = 0.73 to 0.82).

**Conclusion:**

Compared with the general population, people with ID have generally higher overall levels of chronic disease and greater primary care use. Ensuring access to high-quality chronic disease management, especially for epilepsy and mental illness, will help address these greater healthcare needs. Continuity of care and longer appointment times are important potential improvements in primary care.

## INTRODUCTION

People with intellectual disability (ID) (used in this article in preference to the term ‘learning disability’ except where reference is made to specific initiatives such as the Quality and Outcomes Framework [QOF]) are known to have greater healthcare needs with high levels of premature mortality.^1^,^2^ The 2013 confidential inquiry into premature deaths of people with ID in England reported a greater burden of potentially avoidable deaths that may be prevented with good-quality health care.^2^

Concerns over the quality of health care received by people with ID have led to a number of initiatives in primary care in the past 10 years. These include: the adoption of learning disability as a clinical domain in the QOF in 2006; an annual Health Check Scheme introduced in 2009; and the adoption of ID as a clinical priority by the Royal College of General Practitioners in 2010. In addition, all primary care is required by statute to make reasonable adjustments to ensure that the needs of people with ID are met.^3^

Despite these initiatives, there is a continuing paucity of population-based information on the health of people with ID because their experiences are not routinely reported in national primary care, hospital, and mortality data. Specifically, comprehensive primary care information on the healthcare needs and service use of people with ID in the UK is limited, and the need to improve the available data has been recently reinforced.^4^

In this study, the authors used the data from a large primary care database in England to describe chronic disease, comorbidity, disability, and general practice use for adults with ID, and compare these to the general population. Specifically, consultation length and continuity of care were examined because they are potentially important adjustments for improving primary care experience for people with ID.

## METHOD

### Data source

The Clinical Practice Research Datalink (CPRD) is a large, validated primary care database that has been collecting anonymous patient data from participating UK general practices since 1987.^5^ It includes a longitudinal medical record for all registered patients.

### Identification of patients with intellectual disability and matched controls

To identify people with ID, the authors searched for any code used by the QOF for learning disability and codes for conditions usually associated with ID, such as chromosomal and metabolic disorders. This approach identified 21 859 adults (aged ≥18 years) registered in 451 English practices for at least 1 day between 1 January 2009 and 31 March 2013. These individuals were matched based on age and sex from the same practice, with up to seven controls with no record of ID.

**How this fits in**

Although a number of initiatives in primary care have addressed the need to improve the health of people with intellectual disability (ID), there is limited information on their healthcare needs and general practice use. Practices, for their part, are expected to make reasonable adjustments to improve access to care for people with ID. Additionally, the high prevalence of epilepsy and severe mental health problems in people with ID requires effective access to specialist advice. Improving continuity of care and access to longer appointments are therefore important potential improvements in primary care. The results of this study will be helpful in planning and modifying general practice to meet the needs of people with ID and address concerns over the high level of potentially avoidable mortality.

This cross-sectional analysis reports on a subset of 408 practices that were providing high-quality data on 1 January 2012. A total of 14 751 people with ID aged 18–84 years who had been registered for at least 30 days on the 1 January 2012 date were included, along with 86 221 matched controls.

### Recording of chronic disease and disability

The QOF disease registers from the UK general practice contract were used to define chronic disease.^6^ For each condition, the authors searched for the presence of any Read Code in the medical record up to 1 January 2012 to allow the description of prevalence or, more precisely, cumulative incidence. For asthma, epilepsy, and hypothyroidism, in line with the QOF definitions, a recent prescription was also required to give a measure of period prevalence. Severe mental illness was subdivided into schizophrenia and affective disorder; anxiety was defined as an additional condition.

For disability, the authors identified whether an assessment of mobility, continence (after age 12 years), and hearing was ever recorded by 1 January 2012 and whether a problem was noted. For vision, behavioural problems, and constipation, the authors identified recording of a problem ever having occurred.

The authors searched for any evidence that people with ID were living in a communal setting by looking for specific Read Codes or the presence of three or more people with ID with the same address flag, indicating that they were living at the same address.

### Definition of consultation

The aim was to identify unique events where the patient was seen or telephoned by a doctor or nurse. To achieve this in the CPRD, the search was restricted to events where the consultation type (for example, surgery consultation) and staff member (for example, senior partner) met the study’s definition, excluding administrative events and repeat prescribing. For patients with ID, consultations on days where a health check was recorded were excluded.

For face-to-face consultations with a doctor, consultation length was classified into standard (1–10 minutes) and long (>10 minutes), excluding a small number of zero-length consultations. As each clinician has a unique identifier on the system, continuity of care could be estimated by calculating the highest proportion of doctor consultations with the same doctor. A cut-off of >50% was used to summarise continuity; if a patient had a total of five consultations, they would need at least three with the same doctor to achieve this. Although other indices of continuity have been proposed,^7^ this summary has the advantage of being largely independent of number of consultations.

### Statistical analysis

Depending on the outcome, prevalence (PR), odds (OR), or relative risk (RR) ratios between patients with ID and their matched controls were calculated using conditional Poisson and logistic models (Stata version 13). For PRs, Poisson models were fitted with robust error-variance corrections to provide reliable estimates.^8^ Where the outcome was the number of consultations over the previous year, an offset for the number of registered days was added to the Poisson model, to allow for patients who had been registered for less than a year. In the consultation analyses, the data were further adjusted for comorbidity using a weighted score of QOF conditions.^6^ For analyses on consultation length and continuity, the data were also adjusted for the total number of consultations.

## RESULTS

### Characteristics of people with intellectual disability

The ID group had an average age of 42.1 years (standard deviation 15.7) and 57.9% were male (Table 1). Based on the total registered population of the included practices, the estimated prevalence of identified ID was 54 per 10 000 patients; 87.2% of the sample were on their practices’ QOF registers for ID.

**Table 1.** Characteristics of adults with intellectual disability

**Characteristic**	***n***	**%**
All^a^	14 751	100.0
Female	6216	42.1
Male	8535	57.9

**Age, years**		
18–34	5365	36.3
35–54	6041	41.0
55–84	3345	22.6

On QOF LD register	12 862	87.2
Down’s syndrome	1571	10.7
Autism spectrum disorder	1512	10.3

Communal setting	3138	21.3

**Registered, years**		
<1	1037	7.0
1–5	2945	20.0
>5	10 769	73.0

a Registered on 1 January 2012 for at least 30 days.

LD = learning disability. QOF = Quality and Outcomes Framework.

About 1 in 10 of the patients with ID was recorded as having Down’s syndrome. Similarly, 1 in 10 had an additional diagnosis of autistic spectrum disorder. About one-fifth of patients with ID (21.3%) were identified as living within a communal setting.

### Chronic disease prevalence

The pattern of chronic disease is summarised in Table 2. Compared to general population controls, people with ID had a markedly higher prevalence of recorded epilepsy (18.5%, PR 25.3, 95% confidence interval [CI] = 23.29 to 27.57), severe mental illness (8.6%, PR 9.1, 95% CI = 8.34 to 9.92), and dementia (1.1%, PR 7.5, 95% CI = 5.95 to 9.49). In communal settings, the prevalence of epilepsy (27.8%) and severe mental illness (12.6%) was higher (data not shown).

**Table 2.** Prevalence of chronic disease

**Disease**	**Intellectual disability (*n*= 14 751)**		**Controls (*n*= 86 221)**		**Prevalence ratio (95% CI)**
	
	***n***	**%**	***n***	**%**	
Epilepsy^a^	2731	18.5	633	0.7	25.33 (23.29 to 27.57)
Severe mental illness	1266	8.6	823	1.0	9.10 (8.34 to 9.92)
Schizophrenia	995	6.8	591	0.7	9.94 (8.99 to 10.99)
Affective disorder	371	2.5	333	0.4	6.66 (5.73 to 7.73)

IHD	244	1.7	2316	2.7	0.65 (0.57 to 0.74)
Heart failure	121	0.8	324	0.4	2.26 (1.84 to 2.78)
Stroke and TIA	267	1.8	944	1.1	1.74 (1.52 to 1.98)
Atrial fibrillation	122	0.8	821	1.0	0.91 (0.75 to 1.09)
Hypertension	1583	10.7	10 416	12.1	0.93 (0.89 to 0.98)
Peripheral vascular disease	61	0.4	423	0.5	0.90 (0.69 to 1.17)
Chronic kidney disease	468	3.2	1746	2.1	1.64 (1.49 to 1.82)
Diabetes	1017	6.9	3786	4.4	1.64 (1.53 to 1.75)
Hypothyroidism^a^	1169	7.9	2649	3.1	2.69 (2.52 to 2.87)

Asthma^a^	1208	8.2	5717	6.6	1.25 (1.18 to 1.33)
COPD	160	1.1	1184	1.4	0.84 (0.71 to 0.99)

Cancer	238	1.6	2090	2.4	0.70 (0.61 to 0.80)

Osteoporosis	246	1.7	822	1.0	1.84 (1.60 to 2.12)
Rheumatoid arthritis	73	0.5	550	0.6	0.82 (0.65 to 1.05)

Dementia	160	1.1	134	0.2	7.52 (5.95 to 9.49)
Depression	2609	17.7	15 179	17.6	1.03 (0.99 to 1.06)
Anxiety (ever)	2398	16.3	12 580	14.6	1.13 (1.09 to 1.18)

**Number of QOF conditions^b^**					
0	6320	42.8	53 856	62.5	–
1	5056	34.3	20 901	24.2	–
2	2138	14.5	7174	8.3	–
≥3	1237	8.4	4290	5.0	–

a Also require recent medication as per QOF definition.

b Excludes anxiety from the above list.

COPD = chronic obstructive pulmonary disease. IHD = ischaemic heart disease. QOF = Quality and Outcomes Framework. TIA = transient ischaemic attack.

People with ID experienced a moderately increased risk of hypothyroidism and heart failure (PR>2.0). Also significantly higher in patients with ID (PR 1.5–2.0), were stroke, diabetes, chronic kidney disease, and osteoporosis. However, the recorded prevalence of ischaemic heart disease and cancer was approximately 30% lower than in the general population.

A count of the number of chronic conditions per patient confirmed the greater likelihood of multiple comorbidities in people with ID, with 22.9% having ≥2 recorded conditions compared with 13.3% of the control group.

### Problems with daily living

Table 3 summarises the prevalence of disability in people with ID: 41.4% of people with ID had a record of mobility status, with 11.4% overall reporting some form of difficulty, compared to very little (<1%) recording in the controls. For hearing and vision, 4.7% of people with ID had a record of bilateral visual loss or low vision, and 8.3% had a record of severe hearing problems; all higher than the control population. Bowel continence problems were recorded for 3.9%, urinary continence problems for 11.9%, and constipation for 22.9%; 14.1% of people with ID had a recorded behavioural problem in the last 5 years. The recording of all these conditions was considerably lower in the control population. Levels of disability were higher among those identified living in communal settings with 21.4% with a recorded mobility problem, 19.9% with a urinary continence problem, and 24.4% with a behavioural problem recorded in the last 5 years (data not shown).

**Table 3.** Prevalence of disability and other problems

	**Intellectual disability (*n*= 14 751)**		**Controls (*n*= 86 221)**		**Prevalence ratio (95% CI)**
	
	***n***	**%**	***n***	**%**	
**Mobility**					
Recorded ever	6111	41.4	753	0.9	47.58 (43.63 to 51.88)
Some difficulty	1677	11.4	418	0.5	24.02 (21.53 to 26.79)

**Vision**					
Bilateral visual loss or low vision	687	4.7	510	0.6	7.86 (7.01 to 8.82)

**Continence (age ≥12 years)**					
Recorded ever	3017	20.5	3199	3.7	5.68 (5.41 to 5.96)
Bowel problem	579	3.9	240	0.3	14.43 (12.39 to 16.80)
Urinary problem	1755	11.9	2663	3.1	4.00 (3.77 to 4.23)

**Hearing**					
Recorded ever	7361	49.9	9403	10.9	4.58 (4.47 to 4.71)
Impairment	2752	18.7	7111	8.3	2.28 (2.19 to 2.37)
Deaf	1220	8.3	2784	3.2	2.59 (2.42 to 2.76)

**Behavioural problems**					
Last year	564	3.8	155	0.2	21.34 (17.86 to 25.50)
Last 5 years	2072	14.1	742	0.9	16.28 (14.97 to 17.71)

**Constipation**					
Ever	3370	22.9	7135	8.3	2.78 (2.68 to 2.88)

### Consultations

Table 4 describes primary care doctor and nurse consultations in 2011 for people with ID; 86.9% of people with ID consulted at least once in the year compared with 72.6% of matched controls. The average number of consultations in 2011 for people with ID was 6.29 compared with 3.89 in controls (RR 1.70, 95% CI = 1.66 to 1.74). These differences were slightly greater for nurse or telephone consultations and less marked for face-to-face doctor consultations. People with ID in communal settings had a slightly higher number of total (7.51) and face-to-face doctor consultations (5.29) (data not shown).

**Table 4.** Consultations in 2011

	**Intellectual disability (*n*= 14 751)**		**Controls (*n*= 86 221)**		**Relative risk ratio**	
		
	***N*/mean**	**%**	***N*/mean**	**%**	**URR (95% CI)**	**ARR^a^ (95% CI)**
**Number of consultations in 2011**						
0	1936	13.1	22 489	27.4	–	–
1–2	3350	22.7	22 473	26.5	–	–
3–5	3697	25.1	20 080	22.8	–	–
6–11	3568	24.2	15 159	16.8	–	–
≥12	2200	14.9	6020	7.0	–	–

**Mean consultations in 2011**						
All	6.29	100	3.89	100	1.70 (1.66 to 1.74)	1.49 (1.47 to 1.53)
Telephone	0.95	15.1	0.44	11.3	2.26 (2.16 to 2.37)	1.87 (1.78 to 1.97)
Doctor (all)	4.45	70.8	2.88	73.9	1.63 (1.59 to 1.67)	1.45 (1.41 to 1.48)
Nurse	1.84	29.2	1.01	26.1	1.91 (1.83 to 2.00)	1.64 (1.56 to 1.71)
Doctor (face-to-face only)	3.65	58.0	2.52	64.7	1.53 (1.50 to 1.56)	1.37 (1.34 to 1.40)

a Adjusted for comorbidity score that used the following weights: atrial fibrillation (1), diabetes (1), stroke and transient ischaemic attack (1), epilepsy (2), heart failure (2), psychosis, schizophrenia, and bipolar affective disorder (2), chronic obstructive pulmonary disease (2), cancer (3), dementia (3).

ARR = adjusted risk ratio. URR = unadjusted risk ratio.

Although people with ID were more likely to have longer doctor consultations during 2011 (51.3% versus 45.1%), the proportion of consultations >10 minutes was lower (34.7% versus 42.2%). This means that, after taking into account the number of consultations in the year, people with ID were less likely to receive a longer consultation (OR 0.73, 95% CI = 0.69 to 0.77) (Table 5). In terms of continuity of care, people with ID were less likely to see the same doctor >50% of the time in 2011 (43.2% versus 49.1%). This difference was consistent across different numbers of total of consultations, and when adjusted for the total number (OR 0.77, 95% CI = 0.73 to 0.82).

**Table 5.** Consultation length and continuity of care for doctor (face-to-face) consultations

**Group/outcome**	**Intellectual disability (*n*= 14 751)**		**Controls (*n* = 86 221)**		**Odds ratio**	
		
	***N*/mean**	**%**	***N*/mean**	**%**	**UOR (95% CI)**	**AOR^a^ (95% CI)**
**Consultation length**						
All, *N*	3.65	100	2.52	100	–	–
Duration missing or zero, mean	0.21	5.9	0.13	5.2	–	–
Standard length (1–10 minutes), mean	2.17	59.5	1.32	52.6	–	–
Long length (>10 minutes), mean	1.27	34.7	1.06	42.2	–	–
Patients with >1 long consultation (>10 minutes), *N*	7566	51.3	38 880	45.1	1.33 (1.28 to 1.39)	0.73 (0.69 to 0.77)

**Continuity of care**						
All, *N*	9167	100	42 135	100	–	–
Patients with >50% of consultations with same doctor,^b^ *N*	3962	43.2	20 673	49.1	0.77 (0.73 to 0.81)	0.77 (0.73 to 0.82)
**Continuity of care by number of consultations**						
>50% with same doctor, 2–5 total consultations only,^c^ *N*	2690	45.6	14 851	49.0	–	–
>50% with same doctor, 6–11 total consultations only,^c^ *N*	975	39.4	4713	48.7	–	–
>50% with same doctor, 12 total consultations only,^c^ *N*	297	37.7	1109	52.1	–	–

AOR = adjusted odds ratio. UOR = unadjusted odds ratio.

a Adjusted for comorbidity and total number of doctor (face-to-face) consultations.

b Regressions restricted to 8677 match sets where case and at least one control has at least two doctor consultations.

c Totals for this sub-analysis: 2–5 consultations (intellectual disability = 5906, controls = 30 332), 6–11 (intellectual disability = 2473, controls = 9675), ≥12 (intellectual disability = 788, controls = 2128).

## DISCUSSION

This cross-sectional study of over 400 English general practices showed that people with ID have generally higher overall levels of chronic disease with greater overall primary care use, and that this need is greatest in people living in communal settings. However, patients with ID were less likely to have longer doctor consultations and had lower continuity of care with the same doctor.

### Strengths and limitations

This is the first systematic description of the healthcare needs and consultation pattern of people with ID in English primary care. The main strength of the study is the inclusion of a large unselected group of patients with ID identified in primary care. As learning disability has been included in the QOF since 2006, most individuals known to services have likely been identified; however, practices may not identify all ID individuals, especially those with mild ID. Practice-matched comparisons with the general population overcome potential variation in practice recording of chronic conditions and consultation access.

The main limitation of this work is the potential for incomplete recording of some characteristics in primary care. For example, the majority of patients with ID are not categorised in terms of severity of their ID. Evidence from process evaluation of health checks suggests that identification of some chronic conditions and healthcare needs is incomplete in adults with ID; therefore, these results should be interpreted as conservative estimates of the extent of need.^9^

The study describes continuity of care with the same clinician (relational continuity) and does not address other aspects of continuity including consistency of clinical management (management continuity), which may also be important.^10^

### Comparison with existing literature

A number of studies in the UK and internationally have described the prevalence of health problems in people with ID. These have shown high levels of comorbidity although comparison of estimated prevalence is difficult due to population selection and disease definition.^11^–^15^ The overall estimate of the relative increase in consultations in this study is very similar to a Dutch primary care study.^11^

The 18.5% prevalence of epilepsy recorded in this study is lower than some estimates.^16^ This may partially reflect the application of a fastidious definition requiring current treatment and use of a primary care-based rather than register-based population. There is a concern that epilepsy may be overdiagnosed in people with ID and these more recent findings may represent an improvement in diagnosis.^17^ The high prevalence of mental health problems is consistent with a detailed population-based survey undertaken in Glasgow.^18^

Estimates of physical and sensory disability prevalence are sparse with very limited information from UK studies. Reassuringly, the estimates of severe visual problems in this study are close to the prevalence of blindness reported in a well-conducted Dutch study.^14^ Similarly, recorded prevalence of behavioural problems is similar to earlier regional studies in England and Norway.^19^,^20^

### Implications for research and practice

The findings on prevalence of chronic disease raise concern over inadequate identification of some conditions. Specifically, the low prevalence of ischaemic heart disease is surprising given the high prevalence of risk factors including diabetes, obesity, hypothyroidism, chronic kidney disease, and stroke. Similarly, the lower prevalence of cancer needs further exploration because it may indicate late diagnosis or poorer survival. A potential alternative explanation for these findings is lower rates of smoking and alcohol use among adults with ID, but caution should be used when attributing these findings to this without further evidence.

The effect of ID on health is often characterised as a premature ageing phenomenon. In reality, the pattern of comorbidity in people with ID is more complex, with epilepsy and severe mental illness contributing the main burden of excess chronic disease. Both these conditions present challenges to primary care and require good access to specialist services. A recent qualitative study of GPs in Norway highlighted these challenges and the perceived lack of support in managing patients.^21^

The most novel finding of this study is the characterisation of consultation patterns in general practice. The higher consultation rate in primary care is contrary to existing UK data, which suggested a lower consultation rate among people with ID.^22^ The higher rate reported in this study is not explained by the higher prevalence of conditions included in the QOF. This means that the resource implications of caring for people with ID are unlikely to be met through remuneration or systems developed for the QOF. In addition, the high levels of need and use by patients in communal establishments will lead to variable demands on practices depending on local variations in the density of communal establishments.

A key expectation on practices is that they make reasonable adjustments for people with ID and two important potential adjustments are increased consultation times through double appointments and enhanced continuity of care.^23^ The analysis of consultation length in this study provides a mixed picture with a slightly greater likelihood of a longer consultation during the year, but this is reversed when the higher overall likelihood of consultation is taken into account. In other words, any given consultation is likely to be shorter on average for a person with ID.

For continuity of care, people with ID were consistently less likely to see the same doctor. It is possible that this may partly reflect a greater propensity to consult for acute problems where an urgent appointment is more important than continuity. The results of this study suggest that practices could take steps to reach similar levels of long appointments and continuity of care as for the general population. This may be achieved by simple flags on computerised primary care records prompting receptionists to offer double appointments where possible and bypass on-call doctor arrangements for specific patients.

In summary, the results of this study will be helpful in planning and modifying general practice to meet the needs of people with ID and address concerns over the high level of potentially avoidable mortality.^24^
